# On Toxic Neutralisations

**Published:** 1894-10-13

**Authors:** Benjamin Ward Richardson


### On Toxic Neutralisations.

By Sir Benjamin Ward Richardson, M.D., F.R.S.

### II.—RECONSTITUTION.

In the last paper on this subject I dealt with the question of isomeric action as influencing the neutralisation of toxic substances within the body. I pass now to the question of fact whether there can be such an art as shall lead the physician to be able, with precision, to prescribe medicinal agents that shall definitely neutralise toxic agents already within the body, and shall so prove direct remedies of diseases due to toxic substances.

The difficulties lying in the way of this consummation are many. The labour required to bring the matter to the test is great because there are always two subjects to be considered—First, the nature of the toxic agent which has to be naturalised; and, secondly, the action of the neutralising agent itself. There is yet a further trouble, if precise knowledge on the two first points could be acquired, namely, the effect of a new compound which, if produced, might in itself, though different in effect from what it was intended to meet, be toxic. The difficulties altogether have largely prevented true scientific investigation on the lines upon which it was started, and have led, mainly to an empirical or pseudo-scientific development in which certain medicines have been prescribed in disease, under the hope, rather than under the knowledge, that they might be neutralising medicines without any clear definition of the manner in which they acted. We have in fact become flooded with remedies invented on this loose principle, and having no true connection with the scientific principle on which the subject was, in the first instance, laid.

At the same time, even through empirical medicine, certain facts have been elicited which indicate the fact of neutralisation without any true exposition of cause and effect. In the action of iodide of potassium in certain forms of syphillitic disease we witness what seems to be a direct neutralisation, and we must admit it was a grand discovery, leading to one of the most practical results in medicine. The author of this discovery was, I believe, the late Dr. Thomas Williams, of St. Thomas's Hospital, who more than half a century ago lived as a genius of observation in physic, carrying on his labours in St. Thomas's Hospital, publishing a work on morbid poisons, and living and dying so unpretentiously as to be forgotten in history. What the poison is that produces the symptoms which mark those stages of syphillis in which the iodide is so useful we do not know. In what manner the iodide destroys the action of the morbid poison we do not know. Which of the elements, iodine or potassium, causes the neutralisation we do not know. This we do know, that we can prescribe iodide of potassium with the all but positive certainty of its curative effect; of the dose that will be required to produce the cure, and the time which will be necessary for the complete curative change. No honest therapeutist ever pretends to be too certain about the action of remedies. He may calculate what compound will be an anæsthetic; how long it will take to produce sleep; how long the sleep will last, and to some extent what will be the after effect. He may know something, from years of practice, about the action of opium, and, with a certain precision, foresee the proper effects from different modes of prescribing it, and from combining it with other remedies. He may calculate with fair precision the action of salicylate of soda in rheumatic disease—and these are all satisfactory knowledges—but there are none so certain as that of iodide of potassium in the cases in which it is adaptable. The chemist has scarcely a more certain reactive agent in his laboratory.

Potassium iodide was naturally a good starting point on which to commence the inquiry as to neutralisation. In some early researches on the synthesis of cataract certain facts came to my knowledge which strengthened this view. It was found that cataract could be produced synthetically by increasing the specific gravity of the blood through a saline substance, such as sugar or common salt. It was found that all the saline chlorides would cause the same condition, as, for instance, chlorides of potassium, of ammonium, and of sodium. They remained and acted as unbroken physical substances in the body; but when iodides were used in their place, when the iodide of potassium, for instance, was compared in action with the chloride of potassium, cataract was not produced. It seemed as if the iodide of potassium was decomposed within the body, and that therefore its property of increasing and sustaining the specific gravity of the blood was destroyed. Most probably this is what occurs in syphillis; the iodide is decomposed within the body, and by recombination with poisonous products already there brings about new combinations or a new combination, from which there is produced that which is curative of the disease without the formation of a new chemical body that is injurious.

In following out the suggestion that was offered by the action of potassium iodide, I made an endeavour in the year 1867 to ascertain if the use of iodides extended beyond the application already named. In order to determine this it was thought necessary to produce certain fixed symptoms with one substance; and, the cause of the symptoms being known, to ascertain if they could be neutralised by another substance within the precincts of the body. The attempt, therefore, was made to ascertain if the effects of strychnine acting upon the body could be controlled by an easily decomposed iodide, such as iodide of ethyl. If such could be effected, in all probability the spasm of ordinary tetanus, which might possibly be produced by the action of an alkaloidal substance abnormally generated, could be met and successfully neutralised.

These experiments were also extended to the neutralising influence of the amyls over spasmodic action, and also in attempts to neutralise the influence of nicotine by reconstitution. I could not honestly say that the researches were very satisfactory in their results in so far as chemical constitution is concerned; but they led, in turn, to important observations on the counteracting physiological effects of one chemical substance over another, a point to which I shall have to direct attention, and which is of great importance.

